# Clinical outcomes of radiotherapy as initial local therapy for Graves’ ophthalmopathy and predictors of the need for post-radiotherapy decompressive surgery

**DOI:** 10.1186/1748-717X-7-95

**Published:** 2012-06-19

**Authors:** Roshan S Prabhu, Lang Liebman, Ted Wojno, Brent Hayek, William A Hall, Ian Crocker

**Affiliations:** 1 Department of Radiation Oncology, Emory University, Atlanta, Georgia, USA; 2 Winship Cancer Institute, Emory University, Atlanta, Georgia, USA; 3 Division of Oculoplastics, Orbital and Cosmetic Surgery, Emory University, Atlanta, Georgia, USA; 4 Radiation Oncology Associates, Richmond, Georgia, USA

**Keywords:** Radiation therapy, Graves’ disease, Orbital radiation, Graves’ ophthalmopathy, Graves’ orbitopathy

## Abstract

**Background:**

The optimal initial local treatment for patients with Graves’ ophthalmopathy (GO) is not fully characterized. The purpose of this retrospective study is to describe the clinical outcomes of RT as initial local therapy for GO and define predictors of the need for post-RT salvage bony decompressive surgery.

**Methods:**

91 patients with active GO and without prior surgery were treated with RT as initial local therapy between 01/1999 and 12/2010, with a median follow-up period of 18.3 months (range 3.7 - 142 months). RT dose was 24 Gy in 12 fractions. 44 patients (48.4%) had prior use of steroids, with 31 (34.1%) being on steroids at the initiation of RT. The most common presenting symptoms were diplopia (79%), proptosis (71%) and soft tissue signs (62%).

**Results:**

84 patients (92.3%) experienced stabilization or improvement of GO symptoms. 58 patients (64%) experienced improvement in their symptoms. 19 patients (20.9%) underwent salvage post-RT bony decompressive surgery. Smoking status and total symptom score at 4 months were independent predictors of post-RT bony decompression with odds ratios of 3.23 (95% CI 1.03 – 10.2) and 1.59 (95% CI 1.06 – 2.4), respectively. Persistent objective vision loss at 4 months post-RT was the most important symptom type in predicting salvage decompression. Chronic dry eye occurred in 9 patients (9.9%) and cataracts developed in 4 patients (4.4%).

**Conclusions:**

RT is effective and well tolerated as initial local therapy for active GO, with only 21% of patients requiring decompressive surgery post RT. Most patients experience stabilization or improvement of GO symptoms, but moderate to significant response occurs in the minority of patients. Smoking status and total symptom severity at 4 months, primarily persistent objective vision loss, are the primary determinants of the need for post-RT salvage bony decompression. Patients who smoke or present with predominantly vision loss symptoms should be advised as to their lower likelihood of symptomatic response to RT and their increased likelihood of requiring post-RT decompressive surgery.

## Background

Graves’ disease is an autoimmune disease characterized by the production of autoantibodies that activate the thyroid stimulating hormone (TSH) receptor. This receptor is primarily located in thyroid tissue, but also has been found to be expressed in the connective and adipose tissue of retrobulbar contents [1]. Graves ophthalmopathy (GO) is characterized by enlargement and inflammation of the retrobulbar contents, primarily the extraocular eye muscles (EOM). Mucinous material is deposited in the EOM, leading to an inflammatory and fibrotic reaction involving fibroblast proliferation and lymphocyte infiltration [2]. Additionally, some patients with GO produce autoantibodies that interact with orbital fibroblasts, leading to the secretion and deposition of collagen and mucopolysaccharides behind the globe that result in increased intraorbital pressure. The combination of both processes produces the typical spectra of GO symptoms, including proptosis, EOM dysfunction leading to diplopia, periorbital edema, and compressive optic neuropathy in more severe instances. Approximately 20 – 25% of patients with Graves’ disease will present with clinically significant GO, although only about 3 – 5% will develop severe disease [3].

Radiation therapy (RT) is an established modality for the treatment of moderate to severe GO. Its mechanism of action is primary through non-specific anti-inflammatory effects, as well by affecting highly radiosensitive infiltrating lymphocytes and possibly reducing fibroblast proliferation and mucopolysaccharide secretion [4]. Other modalities used in the treatment of GO include systemic corticosteroids, surgery, or a combination of therapies. The optimal treatment algorithm for patients without progressive vision loss is not well defined. Previous clinical trials have demonstrated that orbital RT is better tolerated and as effective as oral steroids [5], and that the combination of these methods is more effective than either therapy alone [6,7].

We defined local therapy as any non-systemic intervention directed towards orbital symptoms, *i.e.* RT, surgery, or steroid drops. Oral steroids were considered systemic therapy for orbital symptoms. At our institution, we have adopted the policy of treating patients who have moderate to severe GO with radiation therapy with or without steroids as initial local therapy in order to avoid surgical intervention. The purpose of this retrospective review is to assess the efficacy of RT with or without steroids as initial local therapy for clinically significant GO, report the rate of salvage decompressive surgery using this treatment paradigm, and describe factors that are associated with lack of clinical response and the need for salvage bony decompression.

## Methods

### Patients

We reviewed the medical records of 102 consecutive patients treated in our department for GO between 01/1999 and 12/2010. 3 patients were lost to follow-up prior to 4 months post-RT and were excluded. 8 additional patients had undergone orbital/lid surgery prior to RT and were excluded. This left 91 patients without prior surgery who were treated with RT with or without steroids as initial local therapy. The diagnosis of GO was made prior to referral to our department and patients were generally followed in conjunction with oculoplastic surgeons. GO signs and symptoms were classified into 5 general categories based upon the NOSPECS classification system [8]: soft tissue, proptosis, EOM dysfunction, corneal involvement, and sight loss. However, due to known limitations of the NOSPECS classification system [9], we included additional symptom categories of diplopia, orbital pain, and tearing in our evaluation. Each category, except for tearing, was assigned a score of 0 – 2 based on severity of symptoms, with scores of 0, 1, and 2 representing no symptoms, mild to moderate symptoms, and severe symptoms, respectively. Tearing was assigned a score of 0 (no tearing) or 1 (tearing). Total disease severity score was the cumulative score across all listed categories and ranged from 0 to 13. Follow-up with both radiation oncology and oculoplastic surgery generally occurred at 1 month and 4 months post RT, and then every 6 months thereafter unless clinically indicated at an earlier time point. In the absence of progressive symptoms, a decision to undertake surgery was generally made following the 4 month follow-up visit. For the purpose of this study, GO symptoms were retrospectively scored prior to RT, at 1 month, and at 4 month post-treatment according to the previously described scoring system.

Response to RT was based on category and total severity score differences between the baseline scores and 4 month post-treatment scores, which was calculated as [(baseline score - 4 month score)/baseline score]. Overall response was based on total severity score difference and categorized as follows: score difference < 0% - progression, score difference > 0 to ≤ 10% - no response (NR), 10% to ≤ 33% difference - mild response, > 33% to ≤ 66% difference - moderate response, > 66% difference - significant response. Stabilization or improvement of GO symptoms was defined as an overall response of ≥ 0%. Individual symptom category response was defined as complete (CR) if the symptom completely resolved by 4 months, or as partial (PR) if there was an improvement in symptoms without complete resolution. Institutional review board approval was obtained for this study.

### Treatment

All patients were treated with a standardized approach using linear accelerator based external beam radiation therapy. Patients were immobilized supine on the treatment couch with an Aquaplast mask (WFR/Aquaplast Corp., Wyckoff, NJ) and treatment planning was performed using the Eclipse platform (Varian Medical Systems, Palo Alto, CA). A high resolution, thin slice (0.625 mm slice thickness) CT scan without contrast was obtained for treatment planning. All patients were planned with CT based 3-dimensional conformal planning using multileaf collimation for field shaping. The treatment isocenter was generally placed behind the lens in order to half beam block the anterior divergence and minimize dose to the lens. The clinical target volume (CTV) was contoured and encompassed the retrobulbar contents including the EOM and retrobulbar fat. Other contoured structures included the globes, lenses, optic nerves, and brain. The distance from the CTV to block edge was typically 1.5 to 2 cm, except anteriorly, where the field edge was intentionally set to exclude direct irradiation of the lens. Opposed lateral 6 MV beams were generally used for treatment, with wedges employed to maintain dose homogeneity within the target volume. All patients received 24 Gy given in 12 fractions over a 2.5 to 3 week period prescribed to the 97 – 100% isodose line.

### Statistical analysis

Descriptive statistics were compiled to characterize the patient population. Logistic regression analysis was performed using baseline clinical factors and response to RT to determine predictors of orbital decompression after RT as well as complete steroid taper by 2 months post-treatment in patients using steroids at the initiation of RT. A p-value of ≤ 0.05 was considered significant; all tests of significance were two-sided. Predictors with a p-value ≤ 0.07 in univariate logistic analysis were included in the multivariate model. All analyses were carried out using the SPSS version 19.0 statistical software package (IBM Inc., Armonk, NY).

## Results

### Patient characteristics

Ninety-one patients with a median age of 55 years (range 29 – 84 years) were treated with a median follow-up period of 18.3 months (range 3.7 - 142 months). Sixty-one patients (67%) were followed for at least 1 year post-RT. Sixty-seven patients (73.6%) had previous thyroid ablation treatment, including thyroidectomy in 14 (15.4%), radioactive iodine in 52 (57.1%), and both in 1 patient (1.1%). Forty-four patients (48.4%) had prior use of steroids, with 31 (34.1%) being on steroids at the initiation of RT. The minority of patients (29.7%) were current smokers at the time of RT. Median total symptom score prior to RT was 6 (range 1 – 12). The most common presenting symptoms were diplopia (79.1%), followed by proptosis (71.4%) and soft tissue signs (61.5%). Disease severity was categorized by the number and severity of presenting symptoms in combination with the clinical criteria of the Dean McGee Eye Institute [10]. Most patients presented with mild (30.8%) or moderate GO (61.5%), with severe GO symptoms in only 7 patients (7.7%). See Table 1 for additional patient characteristics.

**Table 1:** Patient characteristics

**Characteristic**	**No.**	**%**
Gender		
Male	21	23.1
Female	70	76.9
Previous thyroid treatment		
None	24	26.4
Thyroidectomy	14	15.4
RAI	52	57.1
Both	1	1.1
Hyperthyroid medication use		
Yes	17	18.7
No	74	81.3
Thyroid disease		
Hyperthyroid	83	91.2
Hypothyroid	7	7.7
Euthyroid	1	1.1
Duration of symptoms prior to RT		
≤ 6 months	36	39.6
6 – 18 months	38	41.8
> 18 months	17	18.7
Prior steroid use		
Yes	44	48.4
No	47	51.6
Steroid use at initiation of RT		
Yes	31	34.1
No	60	65.9
Response to prior steroids		
None	12	13.2
Partial	32	35.2
Complete	0	0
Smoking at initiation of RT		
Yes	27	29.7
No	64	70.3
Disease severity		
Mild	28	30.8
Moderate	56	61.5
Severe	7	7.7
Median age	55 years (range 29 – 84)	

*RAI* = radioactive iodine, *RT* = radiation therapy.

### Symptom response

The vast majority of patients (92.3%) experienced stabilization or improvement of their GO symptoms. Median total symptom score prior to RT, at 1 month, and at 4 months was 6 (range 1 – 12), 4 (range 0 – 11), and 3 (range 0 – 10), respectively. Overall response was NR for 26 patients (28.6%), mild response for 21 patients (23.1%), moderate response in 30 patients (33%), and significant response in 7 patients (7.7%). Seven patients (7.7%) experienced worsening of GO symptoms. Overall, 58 patients (63.7%) experienced a response in their total symptom score by 4 month evaluation. Response to RT by symptom category can be seen in Table 2. Pain, EOM, and tearing symptom categories had the highest CR rates of 65.8%, 27.7%, and 25.5%, respectively. Pain and tearing also had the highest symptom improvement rates (combined CR/PR) of 73.7% and 62.7%, respectively. Objective vision loss and proptosis were the most refractory to RT, with response rates (combined CR/PR) of 37.1% and 41.5%, respectively. Of the 31 patients who were on steroids at the initiation of RT, 23 (74.2%) were able to be tapered completely off by 2 months post-RT. Univariate logistic regression analysis was performed to determine potential predictors of complete steroid tapering by 2 month post-RT in patients on steroids at the initiation of RT. See Table 3 for results of this univariate analysis. Although no factors were statistically significant at the alpha = 0.05 level, gender (female *vs.* male), longer duration of symptoms prior to RT, total symptom score prior to RT (≥6 *vs.* <6), and smoking status did have a trend towards decreased probability of complete steroid taper. Eleven patients (12%) experienced long term complications. Chronic dry eye occurred in 9 patients (9.9%) and cataracts developed in 4 patients (4.4%). Of note, no patients developed RT induced retinopathy.

**Table 2:** Response to RT by symptom category

**Category**	**No. (%)**	**CR No. (%)**	**CR/PR No. (%)**
NOSPECS:			
Soft tissue	56 (61.5)	8 (14.3)	32 (57.1)
Proptosis	65 (71.4)	4 (6.2)	27 (41.5)
EOM dysfunction	47 (51.6)	13 (27.7)	21 (44.7)
Corneal Involvement	0 (0)		
Sight loss	35 (38.5)	7 (20)	13 (37.1)
Diplopia	72 (79.1)	15 (20.8)	38 (52.8)
Orbital pain	38 (41.8)	25 (65.8)	28 (73.7)
Tearing	51 (56)	13 (25.5)	32 (62.7)

*RT* = radiation therapy, *CR* = complete response.

*PR* = partial response, *EOM* = extraocular eye muscles.

**Table 3:** Univariate analysis for successful steroid taper

**Characteristic**	**Odds ratio**	**95% CI**	**p-value**
Age*	1.03	0.95 – 1.1	0.49
Sex (F *vs.* M)	0.21	0.04 – 1.3	0.10
Duration of symptoms prior to RT			
≤ 6 months	reference		
6 – 18 months	0.75	0.1 – 5.6	0.78
> 18 months	0.14	0.02 – 1.3	0.08
Total score at diagnosis			
(≥ 6 *vs.* < 6)	0.13	0.01 – 1.2	0.08
Prior steroid use	0.68	0.06 – 7.2	0.75
Response to prior steroids			
(PR *vs.* none)	0.79	0.24 – 2.6	0.70
Current smoking	0.26	0.05 – 1.4	0.12
Total score at 1 month*	0.93	0.6 – 1.4	0.75

* denotes continuous variable.

*CI* = confidence interval, *F* = female, *M* = male, *PR* = partial response.

### Post-radiation therapy surgery

Forty-eight patients (52.7%) underwent surgery after RT. Nineteen patients (20.9%) underwent bony decompression, 30 patients (33%) underwent lid surgery, and 15 patients (16.5%) underwent EOM recession. One procedure type was performed in 32 patients, with 16 patients undergoing more than 1 procedure type. Median time from the end of RT to decompression, lid surgery, and EOM recession was 6.2 months (range 2.3 – 17), 10.9 months (range 3.2 – 34.4), and 9.7 months (range 5.9 – 27.1), respectively. Univariate logistic regression analysis was performed to determine predictors of post-RT bony decompressive surgery. Potential predictors such as age, gender, prior or concurrent steroid use, smoking, symptom presentation, total symptom response, and symptom category response were analyzed. See Table 4 for results of the univariate analysis. Of all of the symptom categories, only objective vision loss at 4 months was significantly associated with post-RT decompressive surgery. Variables with a p-value ≤ 0.07 in univariate analysis were included in the multivariate model. However, objective vision loss at 4 months was not included due to significant collinearity with total symptom score at 4 months. See Table 5 for the results of the multivariate logistic regression. Smoking status and total symptom score at 4 months were independent predictors of post-RT bony decompression with odds ratios of 3.23 (95% CI 1.03 – 10.2) for current smokers (*vs.* non-smokers) and 1.59 (95% CI 1.06 – 2.4) for each additional symptom point at 4 month evaluation. No patient underwent salvage orbital reirradiation.

**Table 4:** Univariate analysis for post-RT decompressive surgery

**Characteristic**	**Odds ratio**	**95% CI**	**p-value**
Age*	0.97	0.9 – 1.02	0.20
Sex (F *vs.* M)	0.8	0.3 – 2.6	0.71
Duration of symptoms prior to RT			
≤ 6 months	reference		
6 – 18 months	0.79	0.3 – 2.5	0.69
> 18 months	1.08	0.3 – 4.2	0.92
Total score at diagnosis			
(≥ 6 *vs.* < 6)	2.71	0.9 – 7.9	0.07
Disease severity			
Mild	reference		
Moderate	2.78	0.7 – 10.6	0.14
Severe	3.33	0.4 – 25.4	0.25
Prior surgery	3.89	0.2 – 65.2	0.35
Prior steroid use	1.24	0.5 – 3.4	0.68
Response to prior steroids			
(PR *vs.* none)	0.84	0.2 – 4 0	83
Steroids at initiation of RT	1.17	0.4 – 3.3	0.77
Current smoking	3.6	1.3 – 10.3	0.02^#^
Total score at 1 month			
(> 4 *vs.* ≤ 4)	3.03	1.04 – 8.9	0.04^#^
Total score at 4 months*	1.65	1.2 – 2.3	0.003^#^
Symptoms at diagnosis:			
Soft tissue	0.63	0.2 – 1.7	0.37
Proptosis	1.15	0.4 – 3.6	0.81
EOM dysfunction	0.62	0.2 – 1.7	0.35
Objective vision loss	1.59	0.6 – 4.4	0.37
Diplopia	2.63	0.6 – 12.5	0.23
Symptoms at 4 months:			
Soft tissue	1.22	0.4 – 3.5	0.70
Proptosis	2.12	0.6 – 7.1	0.22
EOM dysfunction	2	0.7 – 5.7	0.19
Objective vision loss	4.45	1.5 – 12.9	0.01^#^
Diplopia	1.03	0.4 – 2.9	0.96

* denotes continuous variable, ^#^ denotes statistical significance.

*RT* = radiation therapy, *CI* = confidence interval, *F* = female, *M* = male,

*PR* = partial response, *EOM* = extraocular eye muscle.

**Table 5:** Multivariate analysis for post-RT decompressive surgery

**Characteristic**	**Odds ratio**	**95% CI**	**p-value**
Current smoking	3.23	1.03 – 10.2	0.05^#^
Total score at diagnosis			
(≥ 6 *vs.* < 6)	0.87	0.2 – 3.8	0.85
Total score at 1 month			
(> 4 *vs.* ≤ 4)	1.22	0.3 – 5.6	0.79
Total score at 4 months*	1.59	1.06 – 2.4	0.03^#^

* denotes continuous variable, ^#^ denotes statistical significance.

*RT* = radiation therapy, *CI* = confidence interval.

## Discussion

The optimal initial local treatment for patients with GO is not fully characterized, and various treatment modalities including RT, steroids, and surgery have been studied and found to be effective for symptom palliation and stabilization. At our institution, we have adopted a policy of treating patients with moderate to severe GO with RT with or without steroids as initial local therapy. This paradigm is meant to use initial RT as a surgery sparing modality in order to avoid invasive procedures in those patients who respond to RT. The existing literature concerning the treatment of GO with RT is heterogeneous and varied, both in terms of study design and results, but several trends have emerged. It has been established that RT and steroids have similar efficacy in GO symptom palliation, both primarily affecting soft tissue involvement and EOM dysfunction, but RT is more tolerable and hence should be considered before single agent systemic steroids [5]. There is also a consensus that the combination of RT and steroids is more effective than either modality alone [11]. However, RT as a single agent has not been well established and several studies comparing RT with sham RT found minimal difference between arms, except in response rates for diplopia [12,13]. We sought to describe our response rates with RT as initial local therapy as well as determine predictive factors for the need for salvage orbital decompression.

We demonstrated an overall rate of GO stabilization or improvement of 92.3% with initial RT, which is comparable to other published studies [14]. Response by symptom category demonstrated heterogeneity of treatment response to RT. Orbital pain, EOM dysfunction, and tearing had the most robust responses with CR rates ranging from 25.5% to 65.8%. Pain and tearing were also the most responsive in terms of overall improvement, with combined CR/PR rates ranging from 62.7% to 73.7%. In contrast, objective vision loss and proptosis were the most refractory to RT, with symptom improvement rates (combined CR/PR) of only 37.1% to 41.5%. This pattern of varying response by symptom category is in agreement with other similar published studies [15]. These results indicate that the specific constellation of presenting symptoms may play a role in the choice of initial local therapy, as it is suggested that patients with predominantly vision loss and/or proptosis may not have satisfactory responses to RT.

The prolonged use of systemic steroids is associated with a significantly increased risk of a variety of potential side effects [5]. 31 patients (34.1%) were on steroids at the initiation of RT. A primary goal of RT is to achieve a level of symptom palliation that allows for the complete tapering of steroids prior to the development of toxicity. Of the initial 31 patients, 23 (72.4%) were able to be successfully tapered by 2 months post-RT, prior to any surgical intervention. This is lower than other published studies [10], but our completely tapered rate is solely in response to RT, as opposed to after RT and salvage surgery in other series. We performed a logistic regression analysis to determine predictors for successful steroid taper. Although no factors were statistically significant, gender (female *vs.* male), longer duration of symptoms prior to RT, higher total symptom score at diagnosis (≥6 *vs.* <6), and current smoking trended towards less probability of complete steroid taper by 2 months post-RT. The lack of statistical significance may be due to the small number of patients on steroids at the initiation of RT.

The overall salvage surgery rate after radiation therapy in this series was 52.7%. The rates of bony decompression, EOM recession, and lid retraction repair were 20.9%, 16.5%, and 33%, respectively. This is similar to a series by Gorman *et al.*, where the overall salvage surgery rate was 50%, and the rates of decompression, EOM recession, and lid retraction repair were 19%, 26%, and 43%, respectively [16]. Orbital decompression is an invasive procedure that has reported risks of complications, including facial parethesias, sinusitis, diplopia, and vision loss, as high as 10 - 17% [17]. The use of RT as initial local therapy is an attempt to minimize the need for orbital surgery, specifically orbital decompression. We performed a logistic regression analysis in order to better define predictors of post-RT bony decompression. The symptom category found to be most associated with the need for salvage decompression was objective vision loss at 4 months post-RT. Considering that objective vision loss was relatively refractory to RT with an overall response rate of only 37%, combined with the association between persistent vision loss and the need for decompression, our data suggest that patients presenting with objective vision loss should be advised of their worse prognosis and elevated risk of salvage decompression. The crude rate of bony decompression in patients with vision loss at 4 months was 40%, approximately twice the rate of the entire study population. Conversely, 60% of these patients did not undergo salvage decompression after initial RT, suggesting that the majority of these higher risk patients can still be spared eventual surgical intervention with this treatment paradigm. In addition, it has been reported that previous RT with or without steroids does not adversely affect the outcome of eventual decompressive surgery [18].

A multivariate logistic analysis demonstrated that smoking at the time of RT and total symptom score at 4 months are independently associated with the need for salvage bony decompression. Smoking is not only approximately 1.5 times more prevalent among those with Graves’ disease and 2 times more prevalent among those with GO [2], but current smoking also has a detrimental effect on palliative response to RT [19]. Our study demonstrates that the detrimental effects of smoking extend further to include a significantly increased risk of post-RT decompressive surgery. It is not currently known what time period is required after the cessation of smoking to decrease the risks of reduced response to RT and higher rates of salvage surgery, but our data indicate that patients with GO who are currently smoking should be extensively counseled about these increased risks and attempts should be made to facilitate smoking cessation.

Orbital RT was well tolerated in our series with no patients requiring treatment breaks for acute toxicity. Long term toxicity was manageable with 11 patients (12%) experiencing long term complications. Nine patients developed chronic dry eye, with all patients successfully medically managed. Four patients developed cataracts, all of whom underwent successful cataract removal and lens replacement. These rates of long term complications are similar to other published series [10]. However, these rates may represent an overestimation of RT toxicity as other known factors, such as age, medical comorbidities, and steroid use, can all contribute to this spectrum of long term complications. Importantly, no patients developed radiation retinopathy.

There are a limited number of reports in the literature describing the natural history of GO, but a potential criticism of RT (or surgery) as initial local therapy is the possibility of spontaneous improvement in GO symptoms with conservative management. An observational study of 59 patients with GO with a median follow-up period of 12 months found that 64% of patients experienced spontaneous improvement in their GO symptoms without local therapy or immunosuppressant use [20]. However, the severity of symptoms in this observational study was significantly less than in our study population, and patients in our study with duration of symptoms ≤ 6 months had generally already failed prior observation and/or steroid therapy.

A potential criticism of RT as initial local therapy, especially in patients who have not had a trial course of steroids, is the theoretical increased risk of secondary malignancy. The excess lifetime risk of radiation induced fatal cancer after RT for GO was estimated to be 7 cases per 1000 persons (0.7%) [21]. The excess lifetime risk of any radiogenic carcinoma was estimated to be possibly as high as 1 – 1.4%. However, these estimations were determined with the assumption of an excess lifetime fatal radiogenic cancer risk of 10% per Sievert (Sv). The median age of our study population was 55 years old, with the vast majority of patients over the age of 45 years. The estimated excess lifetime fatal radiogenic cancer risk for patients of this age range is only 6% per Sv, and decreases with older age at the time of RT. This theoretical risk was felt to be acceptable in this patient population with moderate to severe GO symptoms. The duration of follow-up was not sufficient to formally assess risk of secondary malignancy, though no patients did develop a malignancy of the head or neck region during the follow-up period.

## Conclusions

Orbital RT is an established and safe modality for the treatment of GO. Most patients experience stabilization or improvement of GO symptoms, but moderate to significant response occurs in the minority of patients. The response of specific symptom categories to RT is varied, with objective vision loss and proptosis being the most refractory. Most patients are able to completely taper off steroids by 2 months post-RT, although female gender, longer duration of symptoms prior to RT, higher symptom score at diagnosis, and smoking trended towards an association with inability to taper steroids. Persistent objective vision loss at 4 months is the symptom category most predictive of salvage bony decompression. Current smoking and higher 4 month symptom score are independently associated with a significantly elevated probability of post-RT decompressive surgery. Patients who smoke or present with predominantly vision loss symptoms should be advised as to their lower likelihood of symptomatic response to RT and their increased likelihood of requiring post-RT decompressive surgery. RT with or without steroids as initial local therapy is a viable treatment paradigm for the majority of patients presenting with GO.

## Competing interests

No real or potential competing interest exist for any of the authors.

Financial competing interests:

· In the past five years have you received reimbursements, fees, funding, or salary from an organization that may in any way gain or lose financially from the publication of this manuscript, either now or in the future? *Answer: No* · Is such an organization financing this manuscript (including the article-processing charge)? If so, please specify. *Answer: No* · Do you hold any stocks or shares in an organization that may in any way gain or lose financially from the publication of this manuscript, either now or in the future? If so, please specify. *Answer: No* · Do you hold or are you currently applying for any patents relating to the content of the manuscript? *Answer: No* · Have you received reimbursements, fees, funding, or salary from an organization that holds or has applied for patents relating to the content of the manuscript? If so, please specify. *Answer: No* · Do you have any other financial competing interests? If so, please specify. *Answer: No*.

Non-financial competing interests:

· Are there any non-financial competing interests (political, personal, religious, ideological, academic, intellectual, commercial or any other) to declare in relation to this manuscript? If so, please specify. *Answer: No*.

## Authors’ contribution

RSP participated in the design of the study, data collection, statistical analysis, and drafted the manuscript. LL participated in the design of the study, data collection, and reviewed the manuscript. TW participated in data collection and reviewed the manuscript. BH participated in data collection and reviewed the manuscript. WH participated in data collection, statistical analysis, and reviewed the manuscript. IC participated in the conception and design of the study and reviewed the manuscript. All authors read and approved the final manuscript.
